# Public health round-up

**DOI:** 10.2471/BLT.25.010525

**Published:** 2025-05-01

**WHO scales up emergency response in earthquake-hit Myanmar**

On 28 March 2025, two powerful earthquakes struck central Myanmar’s Sagaing region. The World Health Organization (WHO) has provided nearly 100 tonnes of medicines, medical devices and tents so far, and is assisting in coordination and deployment of emergency medical teams to meet the growing and evolving health needs of the nearly 12.9 million people affected.

**Figure:**
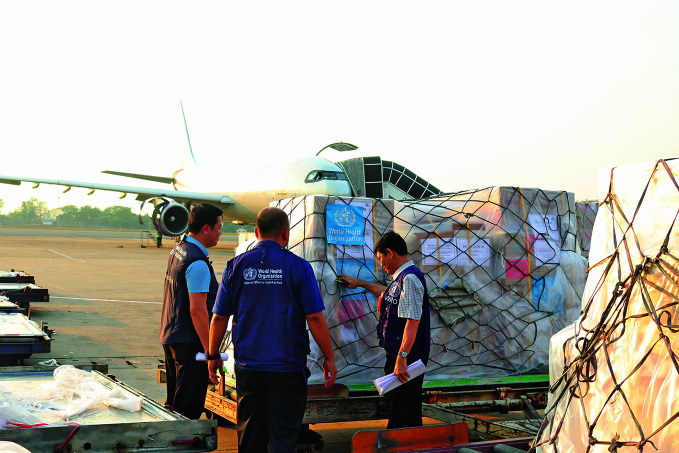
WHO Myanmar/Seng Aung

## Call for global action in Gaza

In a joint statement on 7 April, heads of six United Nations (UN) agencies – including the United Nations Office for the Coordination of Humanitarian Affairs, the United Nations Children's Fund, the World Health Organization (WHO), the World Food Programme, the United Nations Office for Project Services, and United Nations Relief and Works Agency for Palestine Refugees in the Near East – issued an urgent call for global action to protect civilians and ensure humanitarian aid reaches Gaza. For over a month, no commercial or humanitarian supplies have entered the territory, leaving over 2.1 million people without food, water, medicine or safe shelter. 

Over 1000 children have reportedly been killed or injured in just one week following the breakdown of the ceasefire – the highest one-week death toll among children in Gaza in the past year. 

Despite the temporary ceasefire enabling limited aid access, critical supplies remain stalled at border crossings, and conditions are rapidly deteriorating. UN leaders are calling on world leaders to act to ensure the basic principles of international humanitarian law are upheld. 

https://bit.ly/44lC9m7

## Aid cuts threaten progress in maternal health

A new UN report released on World Health Day reveals a 40% global decline in maternal deaths between 2000 and 2023, largely due to improved access to health services. However, this progress is at risk as international aid cuts disrupt vital maternal and newborn care, particularly in fragile and conflict-affected regions.

In 2023, an estimated 260 000 women died from pregnancy or childbirth complications – about one every two minutes. The report underscores how funding shortfalls have led to facility closures, workforce reductions and disrupted supply chains for essential medicines.

The report warns that nearly two thirds of maternal deaths now occur in fragile or conflict-affected settings, where risks are dramatically higher. “While this report shows glimmers of hope, the data also highlight how dangerous pregnancy still is in much of the world today, despite the fact that solutions exist to prevent and treat the complications that cause the vast majority of maternal deaths,” said WHO Director-General Tedros Adhanom Ghebreyesus.

UN agencies urge urgent investment in health systems, midwives and community health workers to reduce avoidable mortality. Improving women’s access to education, reproductive health and care for underlying conditions is also key. 

https://bit.ly/3Ri4AtB

## Global pandemic response test 

WHO brought together over 15 countries and 20 regional health agencies for Exercise Polaris, a two-day simulation testing global coordination in response to a fictional pandemic. The event marked the first test of WHO’s Global Health Emergency Corps, a new framework aimed at improving emergency workforce deployment and cross-border collaboration.

Participating nations activated their national emergency systems to simulate real-time response to a rapidly spreading virus. Over 350 global health experts joined the exercise, working alongside partners including the Africa Centres for Disease Control and Prevention and the Global Outbreak Alert and Response Network.

The simulation emphasized the importance of coordination, trust and speed in global crisis response. WHO provided technical guidance while countries led their own responses under realistic conditions.

“This exercise proves that when countries lead and partners connect, the world is better prepared,” said WHO Director-General Tedros Adhanom Ghebreyesus. “No country can face the next pandemic alone. Polaris shows that global cooperation is not only possible – it is essential.”

https://bit.ly/4cvMzBL

## Invasive fungal diseases

WHO has released its first-ever report addressing the critical lack of medicines and diagnostic tools for invasive fungal diseases, calling attention to a growing public health threat. These infections, which disproportionately affect immunocompromised individuals, are becoming increasingly resistant to available treatments.

Fungal diseases are often misdiagnosed or go undetected, particularly in low- and middle-income countries where access to testing and antifungal medicines is limited. The report on antifungal drugs warns that mortality rates for certain fungal infections can reach up to 88%, yet only four antifungal drugs have been approved globally in the past decade, and just three are in late-stage development.

“Invasive fungal infections threaten the lives of the most vulnerable, but countries lack the treatments needed to save lives,” said Dr Yukiko Nakatani, WHO Assistant Director-General for Antimicrobial Resistance *ad interim*.

The diagnostics report also shows that most tests require sophisticated laboratory infrastructure, making them inaccessible in many regions. WHO is urging investment in safer, more effective treatments, point-of-care diagnostics and research into new drug targets. 

https://bit.ly/4idBEOc

## Healthy cities awards

At the 2025 Partnership for Healthy Cities Summit in Paris, France, Córdoba (Argentina), Fortaleza (Brazil) and Greater Manchester (United Kingdom of Great Britain and Northern Ireland) were honoured for outstanding achievements in preventing noncommunicable diseases. The summit, co-hosted by Bloomberg Philanthropies, WHO, Vital Strategies and the city of Paris, brought together leaders from 61 cities to exchange strategies for advancing local public health.

Córdoba was recognized for its healthy food policy in schools, eliminating sugary drinks and ultra-processed products by 2026. The initiative has already reached 26 schools and 15 000 students. Fortaleza became the first city in Brazil to establish a legal framework for air quality surveillance, including the installation of low-cost pollution sensors to generate health-impact data and guide future environmental policies. Greater Manchester expanded outdoor smoke-free zones and opened its first smoke-free park. The city also developed toolkits and community resources to support the broader adoption of smoke-free environments.

All three cities are part of the Partnership’s Policy Accelerator, which supports the creation and implementation of impactful public health policies. Their achievements reflect the growing role of city governments in shaping healthier urban environments, and serve as models for global action.

https://bit.ly/4j5RSdv

## Disruptions to health systems globally

WHO has issued a warning about widespread disruptions to health systems globally, following sudden cuts and suspensions in official development assistance for health. A rapid assessment conducted in March–April 2025 across 108 WHO country offices – mainly in low- and lower-middle-income countries – revealed that 70% of these countries are already experiencing significant interruptions in health services. These include setbacks in emergency preparedness, disease surveillance, routine health services and humanitarian aid.

Crucially, 24% of countries reported that budget reductions are leading to increased out-of-pocket costs, disproportionately affecting poor and vulnerable populations. Shortages of essential medicines and job losses among health workers were also flagged in over half of the surveyed countries. The scale of disruption mirrors that of the peak COVID-19 pandemic period in some regions.

Despite these challenges, WHO notes that many countries are attempting to reallocate domestic resources and seek alternative funding. Director-General Tedros Adhanom Ghebreyesus emphasized the urgent need for international support while also highlighting a shift towards more sustainable, self-reliant health systems. WHO will continue to monitor developments and coordinate with global partners to mitigate the impact and guide future interventions.

https://bit.ly/4luhwdz

## Draft pandemic agreement

After over three years of negotiation, WHO Member States have finalized a draft pandemic agreement to be considered at the World Health Assembly in May 2025. The agreement aims to strengthen global cooperation on pandemic prevention, preparedness and response.

Established in December 2021, the Intergovernmental Negotiating Body (INB) held 13 formal and multiple informal sessions to craft the proposal. Key elements include a pathogen access and benefit-sharing system, equitable access to health tools, technology transfer, a skilled global health workforce and enhanced health system resilience.

The draft affirms national sovereignty over public health decisions and ensures WHO will not have authority to mandate national policies such as lockdowns or vaccine requirements.

The WHO Director-General hailed the outcome as a historic achievement and a testament to global unity. INB co-chairs Ms Precious Matsoso and Ambassador Anne-Claire Amprou emphasized the agreement’s focus on equity, solidarity and collective security.

https://bit.ly/3G5PJ3c

**Cover photo**

Sudanese refugees in Adre, Chad, unload trucks with food bags.

**Figure:**
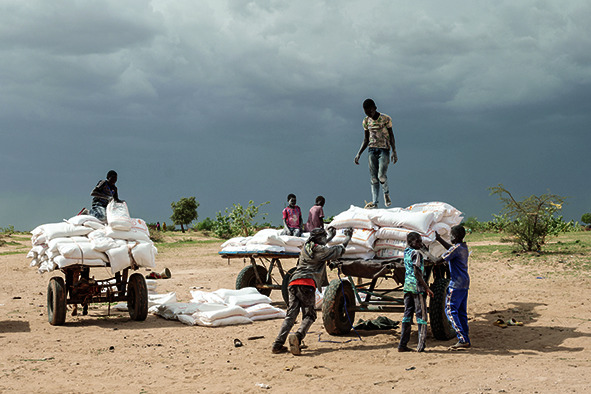
(WHO/Nicolò Filippo Rosso)

**Looking ahead**

12–18 May. 8^th^ UN Global Road Safety Week. https://bit.ly/42I3uO9

19–27 May. Seventy-eighth World Health Assembly. Geneva, Switzerland. https://bit.ly/3FE0auj

31 May. World No Tobacco Day 2025. https://bit.ly/42I3yNT

